# The Critical Role of Nutritional and Procedural Factors in CTO-PCI Patient Prognosis

**DOI:** 10.3390/life16020338

**Published:** 2026-02-15

**Authors:** Gürkan Karaca, Ahmet Ekmekci, Ali Kimiaei, Seyedehtina Safaei, Aziz İnan Çelik, Metin Çağdaş

**Affiliations:** 1 Department of Cardiology, Faculty of Medicine, Bahçeşehir University, Istanbul 34353, Türkiye; karacagurkan@gmail.com (G.K.); ahmetekmekci@yahoo.com (A.E.); tinasafaei@outlook.com (S.S.); 2 Department of Cardiology, VM Medical Park Maltepe Hospital, Istanbul 34846, Türkiye; 3 Hatay Mustafa Kemal University Hospital, Serinyol, Hatay 31115, Türkiye; 4 Department of Cardiology, Kocaeli Gebze Fatih State Hospital, Kocaeli 41400, Türkiye; azizinanmd@hotmail.com; 5 Department of Cardiology, Kocaeli City Hospital, İzmit 41060, Türkiye; metin-cagdas@hotmail.com

**Keywords:** chronic total occlusion (CTO), percutaneous coronary intervention (PCI), procedural complexity, Prognostic Nutritional Index (PNI), risk stratification

## Abstract

(1) Background: Chronic total occlusion percutaneous coronary intervention (CTO-PCI) is a complex, high-risk procedure compared to standard percutaneous coronary intervention (PCI). Scoring systems such as the Japanese Chronic Total Occlusion (J-CTO), European Chronic Total Occlusion (EuroCTO), and Prospective Global Registry for the Study of Chronic Total Occlusion Intervention (PROGRESS-CTO) evaluate lesion difficulty and predict outcomes. Nutritional status, measured by the Prognostic Nutritional Index (PNI), may also affect procedural success and long-term survival. The objective of this study was to evaluate the combined impact of procedural complexity and nutritional status on the clinical outcomes of patients undergoing CTO-PCI. (2) Methods: We analyzed 118 patients undergoing CTO-PCI between May 2021 and March 2022. Procedural complexity was assessed using the J-CTO, EuroCTO, and PROGRESS-CTO scores, while nutritional status was evaluated using the PNI. Primary outcomes included all-cause mortality and repeat revascularization, which were analyzed using Cox proportional hazards regression and Kaplan–Meier survival analyses. (3) Results: Adverse outcomes occurred in 25 patients (mortality: 17; revascularization: 8). Patients with adverse outcomes had significantly lower left ventricular ejection fraction (LVEF) (46 ± 13.7% vs. 52.1 ± 10.5%, *p* < 0.001), lower PNI (*p* < 0.001), and higher J-CTO, EuroCTO, and PROGRESS-CTO scores (all *p* < 0.05). A PNI cut-off value of 46 predicted mortality with a sensitivity of 70.6% and specificity of 75.2% (area under the curve [AUC] = 0.739, *p* = 0.001). Multivariable analysis identified LVEF (hazard ratio [HR] 0.966, *p* = 0.036), J-CTO score (HR 1.598, *p* = 0.027), and PNI (HR 0.925, *p* = 0.022) as independent predictors of mortality. (4) Conclusion: Both procedural complexity and nutritional status significantly influence outcomes following CTO-PCI. Incorporating PNI together with procedural complexity scores into pre-procedural assessments may enhance risk stratification and optimize patient management.

## 1. Introduction

Chronic total occlusion (CTO) of the coronary arteries is one of the most challenging scenarios in interventional cardiology, accounting for nearly 20% of cases in patients undergoing invasive coronary angiography [1]. Percutaneous coronary intervention (PCI) for CTO, referred to as chronic total occlusion percutaneous coronary intervention (CTO-PCI), is a highly complex procedure due to complete coronary artery occlusion lasting longer than three months [2]. Successful revascularization through CTO-PCI has been shown to improve symptoms, reduce the need for subsequent coronary artery bypass grafting (CABG), and enhance survival. However, CTO-PCI is associated with a higher technical failure rate and greater procedural risk than standard PCI [3].

To address these challenges, several procedural scoring systems have been developed to predict technical difficulty, success rates, and potential complications of CTO-PCI. These include the Japanese Chronic Total Occlusion (J-CTO) score, the European Chronic Total Occlusion score incorporating the CABG, age, stump anatomy, tortuosity, length, and extent of calcification (EuroCTO [CASTLE]) score, and the Prospective Global Registry for the Study of Chronic Total Occlusion Intervention (PROGRESS-CTO) score [4,5,6]. These scores assess key factors such as lesion length, calcification, vessel tortuosity, and previous CABG, which are critical in selecting techniques and strategies for successful CTO revascularization [4,5,6]. However, patient-related factors such as nutritional and immunological status are also gaining recognition as important predictors of outcomes in high-risk cardiovascular procedures [7].

The Prognostic Nutritional Index (PNI), which integrates serum albumin concentration and total lymphocyte count, has emerged as a practical tool for assessing patients’ nutritional and immunological status [8]. Low PNI has been associated with increased mortality in various clinical settings, including cardiovascular diseases [8,9]. In the context of CTO-PCI, the potential impact of PNI on procedural outcomes and long-term survival is not yet fully understood.

The objective of this study was to evaluate the combined prognostic impact of procedural complexity, measured by J-CTO, EuroCTO, and PROGRESS-CTO scores, and nutritional status, assessed via the PNI, on adverse outcomes (mortality and revascularization) in patients undergoing CTO-PCI.

## 2. Methods

### 2.1. Study Design and Population

This retrospective observational study included 118 consecutive patients who underwent percutaneous coronary intervention (PCI) for chronic total occlusion (CTO). Patient data were obtained from the hospital’s electronic medical records. To be eligible for inclusion, patients were required to have a diagnosis of chronic total occlusion, defined as complete (100%) occlusion of a coronary artery persisting for at least three months, as confirmed by angiographic evidence [2]. Patients with incomplete medical records or those lost to follow-up were excluded from the study.

### 2.2. Data Collection

Patients were divided into two groups based on clinical outcomes: outcome absent (no mortality or revascularization during follow-up) and outcome present (either mortality or need for revascularization). The primary outcome was all-cause mortality, and the secondary outcome was the requirement for target vessel revascularization during follow-up. This study was conducted in compliance with the Declaration of Helsinki and was approved by the local Institutional Ethics Committee on 10 August 2023 (approval number 4572).

### 2.3. CTO Procedural Scoring

Three established scoring systems were used to assess the complexity of chronic total occlusion percutaneous coronary intervention (CTO-PCI) procedures:

J-CTO Score (Japan Chronic Total Occlusion Registry): The J-CTO score, developed by the Japan Chronic Total Occlusion Registry, assesses the likelihood of successfully crossing a CTO lesion within 30 min by evaluating five key factors: blunt proximal cap, angulation greater than 45°, occlusion length exceeding 20 mm, calcification within the lesion, and prior unsuccessful PCI attempts [4].

EuroCTO (CASTLE) Score: The EuroCTO score, also known as the Coronary Artery Bypass Grafting history, Age, Stump anatomy, Tortuosity, Length of occlusion, and Extent of calcification (CASTLE) score, incorporates both patient- and lesion-related characteristics to assess chronic total occlusion difficulty. These include a history of coronary artery bypass grafting (CABG), age 70 years or older, stump morphology (blunt or undetectable), severe or indiscernible vessel tortuosity, lesion length of 20 mm or more, and significant calcification [5].

Prospective Global Registry for the Study of Chronic Total Occlusion Intervention (PROGRESS-CTO) Score: This scoring system includes four distinct predictors for assessing the complexity of CTO lesions: proximal cap ambiguity, moderate to severe vessel tortuosity, CTO in the Circumflex artery, and the lack of interventional collateral channels [6].

The procedural strategy was left to the discretion of the treating physician, including the choice of approach (antegrade or retrograde), wire selection, microcatheter use, and the decision to use drug-eluting stents. Optimal medical therapy was provided in accordance with current clinical guidelines. While baseline medications (Table 1) reflect admission status, all patients were discharged on guideline-directed medical therapy, including dual antiplatelet therapy (aspirin plus clopidogrel, ticagrelor, or prasugrel) and high-intensity statins, unless contraindicated. The duration of dual antiplatelet therapy was determined based on individual patient factors.

### 2.4. Nutritional and Immunological Status

The Prognostic Nutritional Index (PNI), a validated marker of nutritional and immune status, was calculated using the following formula [10]:

PNI = (10 × serum albumin (g/dL)) + (0.005 × total lymphocyte count (per mm^3^))

The PNI reflects serum albumin levels, indicating nutritional status, and the absolute lymphocyte count, reflecting immunological status [10]. Lower PNI values suggest poorer nutritional and immune function, which has been linked to worse clinical outcomes in cardiovascular disease [10].

### 2.5. Outcome Measures

The study focused on hard endpoints, defined as the most definitive indicators of long-term prognosis. The primary outcome was all-cause mortality, and the secondary outcome was the need for target vessel revascularization during follow-up. These specific endpoints were selected due to their robust availability in retrospective records and their critical clinical significance. Follow-up data were collected from medical records, and patients were monitored for a median period of 13.5 months (IQR 8–23 months). Outcomes were assessed using Kaplan–Meier survival analysis.

### 2.6. Statistical Analysis

Data analysis was performed using Statistical Package for the Social Sciences (SPSS) software version 21.0 (SPSS Inc., Chicago, IL, USA). The normality of continuous variables was assessed using the Kolmogorov–Smirnov test. Descriptive statistics for normally distributed data were reported as mean ± standard deviation, while non-normally distributed data were presented as median (interquartile range). Categorical variables were expressed as frequencies and percentages. The Mann–Whitney U test was used to compare non-normally distributed continuous variables between groups, and categorical variables were compared using either Fisher’s exact test or the Chi-squared test. For continuous variables with a normal distribution, the Independent Samples *t*-test was applied. Statistical significance was defined as a *p*-value < 0.05. Cox regression analysis was conducted to identify independent predictors of mortality, with results reported as hazard ratios (HRs) and 95% confidence intervals (CIs). Survival analysis was performed using the Kaplan–Meier method, and differences in survival outcomes were evaluated using log-rank tests.

## 3. Results

The study comprised 118 patients with a mean age of 63.1 ± 11.2 years, of whom 28.8% were female. Patients were categorized into two groups based on outcome status (outcome absent vs. outcome present). Table 1 summarizes the demographic and clinical characteristics of both groups. No statistically significant differences were observed between the groups with respect to sex, age, diabetes mellitus, hypertension, dyslipidemia, chronic obstructive pulmonary disease, or smoking status.

However, left ventricular ejection fraction (LVEF) was significantly lower in the outcome-present group compared with the outcome-absent group (46 ± 13.7% vs. 52.1 ± 10.5%, *p* < 0.001). Procedural complexity scores were also analyzed. The European Chronic Total Occlusion Coronary Artery Bypass Grafting, Age, Stump anatomy, Tortuosity, Length of occlusion, and Extent of calcification (EuroCTO [CASTLE]) score, the Prospective Global Registry for the Study of Chronic Total Occlusion Intervention (PROGRESS-CTO) score, and the Japanese Chronic Total Occlusion (J-CTO) score were all significantly higher in the outcome-present group (*p* < 0.001, *p* = 0.012, and *p* < 0.001, respectively).

Laboratory analyses revealed that serum albumin levels were significantly lower in the outcome-present group [3.8 (3.4–4.0) vs. 4.0 (3.7–4.1), *p* = 0.011]. Similarly, hemoglobin levels were lower in the outcome-present group [12.0 (11.2–13.4) vs. 13.2 (11.6–14.4), *p* = 0.02]. The Prognostic Nutritional Index (PNI) was also significantly lower in patients with adverse outcomes.

As defined in Section 2, adverse outcomes included all-cause mortality and repeat revascularization. Overall, adverse outcomes occurred in 25 patients, including 17 cases of all-cause mortality and 8 cases of repeat revascularization.

**Figure 1:**
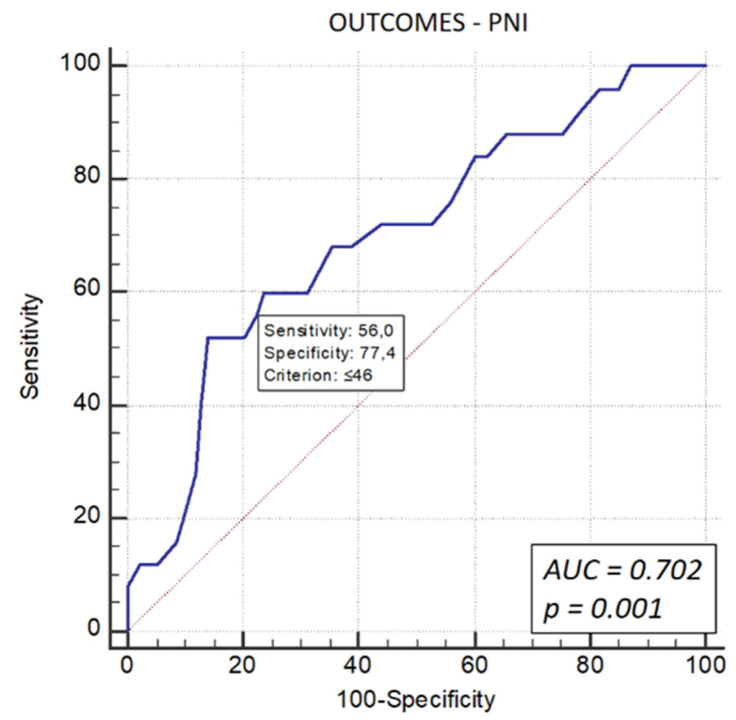
ROC curve analysis for the Prognostic Nutritional Index (PNI) in predicting the composite outcome. The analysis identified a PNI cut-off of 46, which predicted adverse outcomes (mortality + revascularization) with a sensitivity of 56% and specificity of 77.4% (AUC = 0.702, *p* = 0.001).

Receiver operating characteristic (ROC) curve analysis was performed to predict the composite endpoint (all-cause mortality and revascularization) and mortality alone. The analysis yielded an optimal PNI cut-off value of 46. For the composite endpoint, sensitivity was 56%, and specificity was 77.4% (AUC: 0.702, 95% CI: 0.610–0.782, *p* = 0.001) (Figure 1).

**Figure 2:**
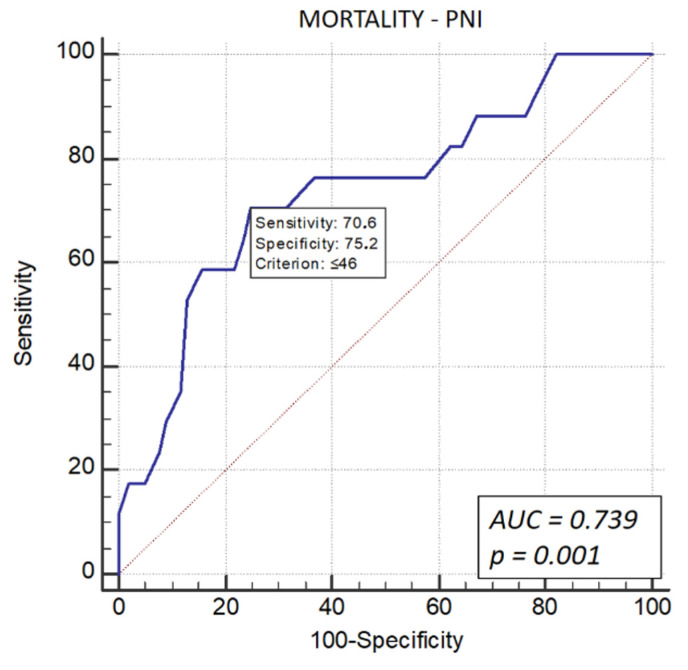
ROC curve analysis for the Prognostic Nutritional Index (PNI) in predicting all-cause mortality. The PNI demonstrated stronger predictive power for mortality alone, with a cut-off of 46 yielding a sensitivity of 70.6% and specificity of 75.2% (AUC = 0.739, *p* = 0.001).

For mortality prediction, sensitivity was 70.6%, and specificity was 75.2% (AUC: 0.739, 95% CI: 0.651–0.816, *p* = 0.001) (Figure 2).

Table 2 summarizes the results of both univariable and multivariable Cox regression analyses for mortality-related factors.

**Figure 3:**
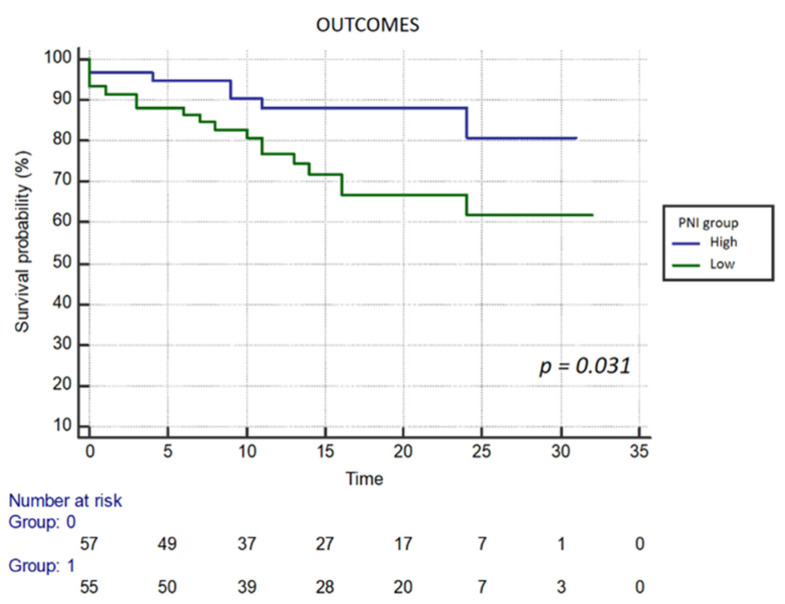
Kaplan–Meier survival analysis for the composite outcome. Patients in the Low PNI group (green line) experienced significantly lower event-free survival rates compared to the High PNI group (blue line) over the follow-up period (log-rank *p* = 0.031).

**Figure 4:**
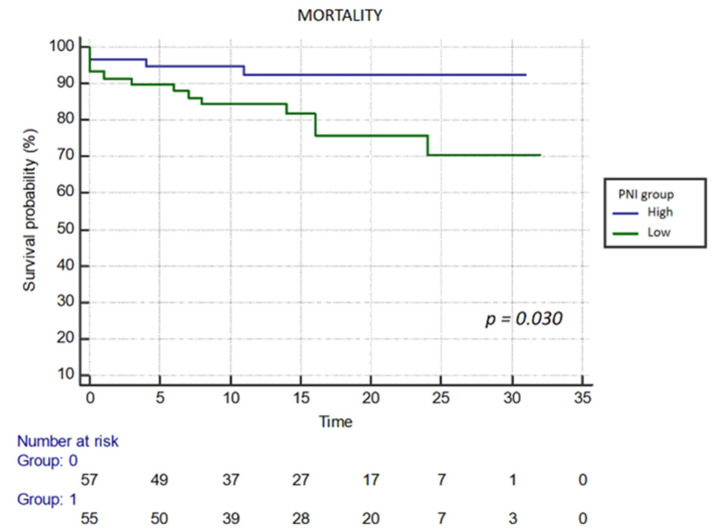
Kaplan–Meier survival analysis for all-cause mortality. The Low PNI group (green line) was associated with significantly higher mortality rates compared to the High PNI group (blue line) (log-rank *p* = 0.030).

Additionally, Kaplan–Meier analysis was performed to evaluate outcomes and mortality based on the PNI group (high or low PNI). The table highlights significant associations with mortality for LVEF (HR 0.966; 95% CI 0.936–0.998; *p* = 0.036), J-CTO score (HR 1.598; 95% CI 1.055–2.420; *p* = 0.027), and PNI (HR 0.925; 95% CI 0.865–0.989; *p* = 0.022). Moreover, Kaplan–Meier curves demonstrate notable differences in outcome and mortality associated with the lower PNI group (*p* = 0.031 vs. *p* = 0.030) (Figure 3 and Figure 4).

## 4. Discussion

In this study, we evaluated the impact of nutritional status, measured by the Prognostic Nutritional Index (PNI), and procedural complexity, assessed using established scoring systems—the Japanese Chronic Total Occlusion (J-CTO), European Chronic Total Occlusion (EuroCTO), and Prospective Global Registry for the Study of Chronic Total Occlusion Intervention (PROGRESS-CTO) scores—on clinical outcomes in patients undergoing percutaneous coronary intervention (PCI) for chronic total occlusion (CTO). Our findings demonstrate that both procedural complexity and nutritional status are significant predictors of adverse outcomes, including mortality and the need for repeat revascularization. Specifically, patients with higher J-CTO, EuroCTO, and PROGRESS-CTO scores were more likely to experience adverse outcomes. Furthermore, lower PNI values were strongly associated with increased mortality and poorer overall outcomes. Notably, left ventricular ejection fraction (LVEF), as well as serum albumin and hemoglobin levels, were significantly lower in patients who experienced adverse outcomes, further emphasizing the influence of systemic health on CTO percutaneous coronary intervention (CTO-PCI) success.

The association between lower PNI values and adverse outcomes in our cohort highlights the importance of nutritional and immunological health in determining the success of high-risk cardiovascular procedures such as CTO-PCI. Patients with adverse outcomes exhibited significantly lower albumin and hemoglobin levels, both of which are key components of the PNI and established markers of poor nutritional status. These findings are consistent with previous studies examining the prognostic value of PNI in cardiovascular disease. In a large retrospective study by Özbek et al. involving 516 patients with CTO, significantly lower PNI values were observed in non-survivors compared with survivors (42.41 ± 6.57 vs. 47.87 ± 6.31) over a 48-month follow-up period, underscoring the role of PNI in predicting long-term outcomes [11]. In our study, receiver operating characteristic analysis demonstrated that a PNI cut-off value of 46 predicted both overall adverse outcomes and mortality, with sensitivities of 56% and 70.6%, respectively. Although this value represented the optimal statistical threshold within our cohort, it should be interpreted clinically as a risk indicator rather than a strict boundary, with progressively lower values reflecting increasing vulnerability to adverse events.

The prognostic utility of immune-nutritional indices extends beyond percutaneous interventions to other revascularization modalities. In a large multicenter cohort of 2889 patients, Sun et al. demonstrated that PNI is a robust independent predictor of both short-term and long-term mortality in patients undergoing coronary artery bypass grafting (CABG) [12]. Their findings highlight that nutritional status fluctuates during the perioperative period, and maintaining a favorable PNI is crucial for survival [12]. This parallels our findings in the CTO-PCI population, suggesting that PNI serves as a universal marker for risk stratification in patients requiring complex coronary revascularization.

These results support PNI as a simple yet effective tool for pre-procedural risk stratification in CTO-PCI patients. Beyond mortality prediction, Akbuğa et al. demonstrated a positive correlation between PNI and coronary collateral development (r = 0.168, *p* = 0.026), suggesting its role in vascular adaptation [13]. Similarly, Esenboga et al.’s study of 400 patients identified PNI as an independent predictor of coronary collateral circulation (OR 0.870; 95% CI 0.822–0.922) [14].

Adding to this growing body of evidence, Tunçez et al. recently investigated the Naples Prognostic Score (NPS), a composite marker similar to PNI that integrates albumin, cholesterol, and lymphocyte ratios [15]. They found that a lower NPS, indicative of better immune-nutritional status, was an independent predictor of well-developed coronary collateral circulation in CTO patients [15]. This further reinforces the hypothesis that systemic inflammatory and nutritional homeostasis is essential for vascular adaptation and collateral growth, which are critical compensatory mechanisms in chronic occlusions.

Consistent evidence links nutrition with cardiovascular outcomes: Wada et al.’s large-scale study of 1988 stable coronary patients confirmed PNI’s association with long-term cardiovascular outcomes [8]. Cheng et al. suggested that the connection between malnutrition and poor outcomes might be driven by inflammation, which can accelerate atherosclerosis [16]. Therefore, addressing nutritional deficits before high-risk procedures potentially through nutritional optimization may improve short- and long-term outcomes, particularly in patients with advanced cardiovascular disease.

Our study demonstrated that procedural complexity scores J-CTO, EuroCTO (CASTLE), and PROGRESS-CTO are key predictors of adverse outcomes in CTO-PCI, with each score showing particular strengths in assessing risk. In our cohort, patients with higher scores in all three systems faced greater procedural difficulty and higher complication rates. In particular, the J-CTO score was a strong predictor of technical failure, consistent with the findings of Karacsonyi et al., who reported J-CTO’s high predictive accuracy (AUC, 0.77), making it especially useful for assessing lesion complexity and procedural difficulty [17]. Brinza et al. also recognized the broad utility of the J-CTO score in determining procedural success based on lesion morphology and historical failed attempts, which closely aligns with our observations of its reliability in predicting adverse events during CTO-PCI [18].

While scoring systems like J-CTO allow us to quantify this complexity, they also drive the development of new management strategies. For instance, Acar et al. recently proposed the ‘GITSU’ strategy (Give It Time to Sober Up), a staged PCI approach for highly complex lesions [19]. By deferring stenting to a second session after initial flow restoration, they achieved high success rates with reduced stent lengths, demonstrating how procedural planning, informed by complexity assessment, can mitigate the risks associated with high lesion scores [19].

The EuroCTO and PROGRESS-CTO scores, as well as their value, offer unique perspectives on risk stratification. We found that higher EuroCTO scores, which consider patient-specific factors such as age and previous CABG, were associated with increased mortality and revascularization needs, supporting the findings of AlAshry et al. and Kalogeropoulos et al. that EuroCTO’s comprehensive approach is particularly useful in complex cases [20,21]. PROGRESS-CTO, which emphasizes angiographic factors alone, was less specific in our study, a finding consistent with Karacsonyi et al. and Brinza et al., who also reported moderate predictive power for this score compared with J-CTO and EuroCTO [17,18]. Collectively, these studies suggest that while each scoring system has unique strengths, using them in tandem allows for more precise risk assessment and tailored strategies, which can help interventionalists improve CTO-PCI outcomes through better-informed decision-making.

Left ventricular ejection fraction (LVEF) is commonly recognized as a measure of cardiac function and is frequently utilized in standard clinical practice [22]. Lower LVEF has long been associated with worse outcomes in cardiovascular interventions, as it reflects impaired cardiac function and a greater burden of heart failure [22,23,24]. In our analysis, patients in the outcome-present group had significantly reduced LVEF compared to those without adverse events, reinforcing the notion that reduced systolic function may predispose these patients to higher procedural risk and long-term complications. However, even after adjusting for LVEF in our multivariable Cox regression model, nutritional status (PNI) and procedural complexity (J-CTO) remained independent predictors of mortality, suggesting their prognostic value extends beyond simple cardiac dysfunction. This finding aligns with several recent studies demonstrating the prognostic importance of LVEF in PCI outcomes. A large-scale analysis of 7827 CTO PCI procedures showed that while technical success rates were similar across LVEF groups, patients with lower LVEF (≤35%) experienced higher in-hospital mortality (1.1% vs. 0.3%) and continued to have higher mortality rates during follow-up (4.9% vs. 1.4%) compared to those with preserved LVEF [25]. However, promising data from a study of 839 CTO PCI patients demonstrated that successful intervention could significantly improve LVEF in severely impaired patients, from 29.1% to 41.6% [26]. Moreover, a prospective study further supported these findings, showing that patients with left ventricular dysfunction had significantly higher rates of major adverse cardiac events (HR: 2.07, 95% CI: 1.03–4.16) and cardiac death (HR: 5.49, 95% CI: 1.29–23.3) compared to those with preserved LVEF ≥50% [27]. Similarly, a prospective cohort study of 304 patients undergoing primary PCI for STEMI found that reduced LVEF was significantly associated with increased in-hospital adverse events [28]. Another study suggested that decreased LVEF may increase the risk of stent thrombosis [29]. These findings collectively underscore the crucial role of LVEF as an important predictor of both short- and long-term outcomes in CTO-PCI, while also highlighting the potential benefits of successful intervention even in patients with severely reduced LVEF.

The findings of this study suggest that a more comprehensive approach to CTO-PCI, which includes consideration of both procedural complexity and the patient’s overall health, particularly nutritional status, could be beneficial. While lesion characteristics and technical challenges are well-recognized factors in planning CTO-PCI, our results indicate that nutritional status, as measured by PNI, may also play a role in patient outcomes. Although further research is needed to confirm these findings, incorporating routine PNI assessments into preprocedural evaluations could help identify patients who might benefit from additional nutritional support before undergoing CTO-PCI.

This study has several limitations. Its retrospective, single-center design and relatively small sample size may have introduced unknown confounding factors and reduced the generalizability of our findings, despite analytical adjustments. Second, PNI values were only measured once at baseline, without accounting for potential changes during follow-up. The reliance on electronic medical records could have also introduced potential biases due to incomplete data collection. Notably, this analysis was restricted to mortality and revascularization; data on other major adverse cardiac events (MACE), such as non-fatal myocardial infarction and cerebrovascular events (stroke), were not consistently available in the retrospective dataset and were therefore excluded. Furthermore, while discharge prescriptions were consistent with guidelines, long-term patient adherence to medical therapy could not be systematically verified. Similarly, while CTO lesion complexity was rigorously scored, the specific extent of significant stenosis in non-CTO vessels (multivessel disease) was not separately quantified, which may have influenced revascularization rates. Finally, specific procedural details, such as the brand names and origins of the devices used (guidewires and microcatheters), were not recorded for this analysis, though standard contemporary equipment was utilized.

## 5. Conclusions

In conclusion, both procedural complexity and nutritional status significantly influence clinical outcomes in chronic total occlusion percutaneous coronary intervention (CTO-PCI). Higher Japanese Chronic Total Occlusion (J-CTO), European Chronic Total Occlusion (EuroCTO), and Prospective Global Registry for the Study of Chronic Total Occlusion Intervention (PROGRESS-CTO) scores were associated with greater procedural difficulty and poorer clinical outcomes, while lower Prognostic Nutritional Index (PNI) values were linked to increased mortality and adverse events. These findings highlight the importance of a comprehensive pre-procedural evaluation that integrates both lesion-related complexity and patient-related health status to optimize the management and outcomes of patients undergoing CTO-PCI. From a practical standpoint, the PNI represents a simple, cost-effective, and widely accessible tool for risk stratification. Routine incorporation of PNI assessment may help identify vulnerable patients who could benefit from targeted nutritional optimization strategies before intervention, potentially improving procedural success rates and long-term survival. Future prospective, multicenter studies are warranted to validate these findings and further define the role of nutritional assessment in CTO-PCI risk stratification.

## Figures and Tables

**Table 1:** Comparison of baseline demographic, clinical, and laboratory characteristics between patients.

Variables	Outcome Absent (*n* = 93)	Outcome Present (*n* = 25)	Total (*n* = 118)	*p* Value
Demographics & Comorbidities				
Age (years)	62.3 ± 10.7	66.2 ± 12.6	63.1 ± 11.2	0.147
Gender (Female), *n* (%)	25 (26.9)	9 (36)	34 (28.8)	0.371
Diabetes mellitus, *n* (%)	32 (34.4)	7 (28)	39 (33.1)	0.545
Hypertension, *n* (%)	48 (51.6)	11 (44)	59 (50)	0.499
Dyslipidemia, *n* (%)	35 (37.6)	5 (20)	40 (33.9)	0.098
Smoking, *n* (%)	26 (28)	5 (20)	31 (26.3)	0.422
COPD, *n* (%)	15 (16.1)	6 (24)	21 (17.8)	0.361
Medications				
ACEi/ARB, *n* (%)	43 (46.2)	10 (40)	53 (44.9)	0.578
Beta-blockers, *n* (%)	23 (24.7)	5 (20)	28 (23.7)	0.622
CCB, *n* (%)	18 (19.4)	5 (20)	23 (19.5)	0.942
Diuretics, *n* (%)	31 (33.3)	7 (28)	38 (32.2)	0.612
Statins, *n* (%)	33 (35.5)	4 (16)	37 (31.4)	0.062
Antiplatelets, *n* (%)	37 (39.8)	8 (32)	45 (38.1)	0.477
Procedural & Clinical Scores				
LVEF (%)	52.1 ± 10.5	46 ± 13.7	50.8 ± 11.5	0.025
J-CTO score	1.2 ± 1	1.9 ± 1	1.4 ± 1	<0.001
EuroCTO (CASTLE) score	1.6 ± 0.9	2.6 ± 1.1	1.8 ± 1	<0.001
PROGRESS-CTO score	0.8 ± 0.7	1.3 ± 0.9	0.9 ± 0.8	0.012
Retrograde approach, *n* (%)	4 (4.3)	1 (4)	5 (4.2)	0.947
Laboratory Findings				
Nutritional & Inflammatory				
PNI	49.4 ± 5.2	45.6 ± 5.4	48.6 ± 5.4	0.002
Albumin (g/dL)	4 (3.7–4.1)	3.8 (3.4–4)	4 (3.7–4.1)	0.011
Hemoglobin (g/dL)	13.2 (11.6–14.4)	12 (11.2–13.4)	13.1 (11.4–14.2)	0.020
Lymphocyte count (×10^9^/L)	2 (1.5–2.5)	1.8 (1.2–2.2)	1.9 (1.4–2.4)	0.136
CRP (mg/dL)	3.3 (2.1–5.3)	3.7 (1.8–7.5)	3.4 (2.1–6.4)	0.504
Metabolic & Renal				
Fasting glucose (mg/dL)	114 (98–180)	114 (100–178)	114 (98–180)	0.573
Creatinine (mg/dL)	1 (0.9–1.2)	1 (0.9–1.1)	1 (0.9–1.1)	0.785
LDL-C (mg/dL)	98 (83–119)	105 (80–139)	98.5 (83–125)	0.737
HDL-C (mg/dL)	40.4 ± 9.8	40.9 ± 7.3	40.5 ± 9.3	0.425
Triglyceride (mg/dL)	126 (107–185)	117 (98–168)	125 (105–175)	0.335
Outcomes				
Mortality, *n* (%)	0 (0)	17 (68)	17 (14.4)	<0.001
Revascularization, *n* (%)	0 (0)	8 (32)	8 (6.8)	<0.001
Follow-up (months)	17 (10–24)	8 (1–11)	13.5 (8–23)	<0.001

Note. ACEi: Angiotensin-converting enzyme inhibitors, ARB: Angiotensin receptor blockers, CCB: Calcium channel blockers, MRA: Mineralocorticoid receptor antagonist, CASTLE: Coronary artery bypass graft history, Age (≥70 years), Stump anatomy (blunt or invisible), Tortuosity degree (severe or unseen), Length of occlusion (≥20 mm), and Extent of calcification (severe), PROGRESS-CTO: Prospective Global Registry for the Study of Chronic Total Occlusion Intervention, J-CTO: Multicenter Chronic Total Occlusion Registry of Japan, ALT: Alanine transaminase, AST: Aspartate Transferase, LDL-C: Low-density lipoprotein cholesterol, HDL-C: High-density lipoprotein cholesterol, CRP: C-reactive protein, PNI: Prognostic nutritional index.

**Table 2:** Univariable and multivariable Cox regression analyses for prediction of mortality in patients.

Univariable	*p* Value	HR	95% CI		Multivariable	*p* Value	HR	95% CI	
			Lower	Upper				Lower	Upper
LVEF	0.012	0.961	0.932	0.991	LVEF	0.036	0.966	0.936	0.998
EuroCTO (CASTLE) score	<0.001	2.291	1.541	3.406	EuroCTO (CASTLE) score	-	-	-	-
PROGRESS-CTO score	0.003	1.971	1.256	3.091	PROGRESS-CTO score	-	-	-	-
J-CTO score	0.009	1.675	1.135	2.472	J-CTO score	0.027	1.598	1.055	2.420
Hemoglobin	0.032	0.795	0.644	0.980	Hemoglobin	-	-	-	-
PNI	0.006	0.907	0.846	0.973	PNI	0.022	0.925	0.865	0.989

Note. LVEF: Left ventricular ejection fraction, CASTLE: Coronary artery bypass graft history, Age (≥70 years), Stump anatomy (blunt or invisible), Tortuosity degree (severe or unseen), Length of occlusion (≥20 mm), and Extent of calcification (severe), PROGRESS-CTO: Prospective Global Registry for the Study of Chronic Total Occlusion Intervention, J-CTO: Multicenter Chronic Total Occlusion Registry of Japan, PNI: Prognostic nutritional index.

## Data Availability

Data is contained within the article.
